# p53 mutations define the chromatin landscape to confer drug tolerance in pancreatic cancer

**DOI:** 10.1002/1878-0261.13161

**Published:** 2022-02-11

**Authors:** Carlotta Zampieri, Emanuele Panatta, Vincenzo Corbo, Alessandro Mauriello, Gerry Melino, Ivano Amelio

**Affiliations:** ^1^ Department of Experimental Medicine TOR, University of Rome Tor Vergata Rome Italy; ^2^ University of Verona Italy; ^3^ School of Life Sciences University of Nottingham UK

**Keywords:** cancer epigenetics, chemoresistance, chemosensitivity, chromatin modifications, gemcitabine, SWI/SNF chromatin remodelling complex

## Abstract

Somatic inactivation of p53 (*TP53*) mainly occurs as missense mutations that lead to the acquisition of neomorphic mutant protein forms. p53 mutants have been postulated to exert gain‐of‐function (GOF) effects, including promotion of metastasis and drug tolerance, which generally contribute to the acquisition of the lethal phenotype. Here, by integrating a p53^R270H^‐dependent transcriptomic analysis with chromatin accessibility (ATAC‐seq) profiling, we shed light on the molecular basis of a p53 mutant‐dependent drug‐tolerant phenotype in pancreatic cancer. p53^R270H^ finely tunes chromatin accessibility in specific genomic loci, orchestrating a transcriptional programme that participates in phenotypic evolution of the cancer. We specifically focused on the p53^R270H^‐dependent regulation of the tyrosine kinase receptor macrophage‐stimulating protein receptor (MST1r). MST1r deregulation substantially impinged on drug response in the experimental model, recapitulating the p53^R270H^‐dependent phenotype, and strongly correlated with p53 mutant and aggressive phenotype in pancreatic cancer patients. As cellular plasticity in the final stages of the evolution of pancreatic cancer seems to predominantly originate from epigenetic mechanisms, we propose that mutant p53 participates in the acquisition of a lethal phenotype by fine‐tuning the chromatin landscape.

## Graphical abstract

This study reports that chromatin accessibility is influenced by p53 mutants at the genomic site of macrophage stimulating 1 receptor (a key player of PDAC chemoresistance), promoting the transcription of the gene. This molecular axis influences drug resistance phenotype in PDAC cell lines, and it is a negative prognostic factor in patients. Thus, our work suggests that interaction between genetic and epigenetic events underlies the basis of PDAC evolution.

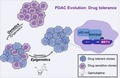

**Abbreviations**
ATAC: assay for transposase‐accessible chromatin
ChIP: chromatin immunoprecipitation
DMEM: Dulbecco's modified Eagle’s medium
ECL: enhanced chemiluminescence
EMEM: Eagle's Minimal Essential Medium
Gem: gemcitabine
GOF: gain of function
GSEA: gene set enrichment analysis
*KPCp53mut*
: *pdx1‐CRE: LSL‐KRASG12D* *;p53mut*
MSP1: MST1, macrophage‐stimulating protein 1
MST1r: macrophage‐stimulating protein receptor
PDAC: pancreatic ductal adenocarcinoma
PE42: paired‐end 42‐bp sequencing reads
PFS: progression‐free survival
Sr.p1: Srebp1
TCGA: The Cancer Genome Atlas
wt: wild‐type

## 1 Introduction

*TP53* gene encodes for the crucial tumour suppressor transcriptional factor p53. While p53 is virtually mutated in every second human cancer, the progression of certain tumour types, including pancreatic ductal adenocarcinoma (PDAC), specifically relies on genetic alteration in TP53 gene [1]. These mutations often generate neomorphic forms of p53 protein, whose gain‐of‐function (GOF) effects seem to display a high degree of context dependency [2, 3]; for example, in different gut microbiome environment p53^R172H^ can exert tumour suppressive or oncogenic functions [4, 5]. Several mechanisms have been suggested to explain mutant p53 GOF: these include interaction with p53 family members [6, 7], alteration in the activity of wild‐type p53 partner transcriptional factors [8, 9, 10] and acquisition of novel binding partners [11]. Perturbation of the physiological signalling probably underlies part of the mutant p53‐dependent phenotype, but a general consensus on whether mutant p53 GOF is mediated by alteration in specific molecular pathways or to less selective perturbations has not been achieved.

PDAC is among the most lethal disease, with a 5‐year survival rate lower than 10% [12]. A model of genetic progression of pancreatic carcinogenesis defines how the disease can advance from a premalignant lesion to form an infiltrating cancer [13]. The progression arises from low‐grade dysplasia carrying KRAS mutation to high‐grade invasive carcinomas, when also TP53 mutations have occurred, with intermediate stages involving inactivation of CDKN2A and SMAD4 [14]. Although this genetic model helps in defining key events in the progression of the disease, it fails to fully explain the evolution of metastatic and drug‐tolerant subclones [15]. Thus, potential phenotypic evolution with an epigenetic basis could contribute to the more advanced stages of tumorigenesis.

Here, we report the integration of a p53^R270H^‐dependent transcriptomic (RNA‐seq) analysis with chromatin accessibility (ATAC‐seq) profiling of pancreatic cancer cells derived from the *pdx1‐CRE: LSL‐KRAS^G12D^;p53^mut^ (KPC^p53mut^)* mouse models. Our data demonstrate the ability of mutant p53 to finely tune chromatin accessibility in specific genomic loci, thus promoting a drug‐tolerant phenotype. We particularly focused on the p53^R270H^‐dependent regulation of the tyrosine kinase receptor, MST1r. The p53^R270H^/MST1r axis appears to crucially impinge on the drug tolerance phenotype of KPC cells and strongly correlate to PDAC patient prognosis. Thus, our data shed light on the ability of mutant p53 GOF to impinge on cancer chromatin landscape, thus orchestrating with extrinsic stressor acquisition of phenotypic plasticity at later stages of cancer progression.

## 2 Materials and methods

### 2.1 Cell culture and transfection

Pancreatic cancer cell lines from mouse models (KPC270 and KPC172)^25^ were grown in Dulbecco's modified Eagle’s medium (DMEM) (Gibco, Waltham, MA, USA), and human (PANC1 and HPAFII) pancreatic cancer cell lines were cultured in DMEM (Gibco) and Eagle’s Minimal Essential Medium (EMEM) (Gibco). Each medium was further supplemented with 10% (FBS, Gibco) and penicillin/streptomycin (2 units·mL^−1^) (Gibco), and the cell lines were maintained at 37 °C under 5% CO2. PANC1 and HPAFII were purchased from ATCC. KPC^R270H^ and KPC^R172H^ were donated by Jennifer Morton. siRNA transfection was performed using Lipofectamine RNAiMAX (Invitrogen, Waltham, MA, USA) with 60 nm Silencer Select Predesigned trp53 (Ambion, siRNA ID s75472, Waltham, MA, USA), 50 nm Silencer Select Predesigned human tp53 (Ambion, siRNA ID s607), Mst1r (Mm_Mst1r_3 FlexiTube siRNA, Qiagen siRNA ID SI01319213, Düsseldorf, Germany) and Silencer Select Negative Control No. 1 siRNA (Ambion).

### 2.2 ATAC‐seq and RNA‐seq

For ATAC‐seq, KPC^R270H^ cells were enzymatically detached using trypsin and 100 000 cells were centrifuged at 500 × ***g*** at 4 °C. The pellet was resuspended in 500 μL of ice‐cold cryopreservation solution – 50% FBS, 40% growth media and 10% DMSO and shipped to active motif to perform RNA‐seq and bioinformatic analyses. The paired‐end 42‐bp sequencing reads (PE42) generated by Illumina sequencing (using NextSeq 500) were mapped to the genome using the BWA algorithm with default settings. Alignment information for each read was stored in the BAM format. Only reads that pass Illumina’s purity filter, align with no more than 2 mismatches and map uniquely to the genome were used in the subsequent analysis. Genomic regions with high levels of transposition/tagging events were determined using the MACS2 peak‐calling algorithm. Since both reads (tags) from paired‐end sequencing represent transposition events, both reads were used for peak‐calling but treated a single, independent read. To identify the density of transposition events along the genome, the genome was divided into 32‐bp bins and the number of fragments in each bin was determined. For this purpose, reads were extended to 200 bp, which is close to the average length of the sequenced library inserts. This information (‘signal map’; histogram of fragment densities) was stored in a bigWig file. The BAM and bigwig files were visualized using UCSC Genome Browser (http://genome.ucsc.edu/index.html), Integrated Genome Browser (http://bioviz.org) and Integrative Genomics Viewer (http://www.broadinstitute.org/igv/). To identify what transcription factor binding motifs were present at those sites, a HOMER‐based analysis of transcription factor binding motifs was performed.

For the RNA‐seq, the RNA was extracted as described below and shipped to Active Motif to perform RNA‐seq and bioinformatic analyses.

### 2.3 Immunoblot analysis

After 48 h of silencing, KPC^R270H^ were treated with 10 nm and 100 nm of gemcitabine hydrochloride (Sigma, cat. no. G6423‐10MG, Burlington, MA, USA) and were collected after 10, 16 and 24 h of treatment. Cells were extracted in RIPA buffer (50 mm Tris/HCl, pH=7,5; 0,5% NP‐40; 250 mm NaCl; 1 mm EDTA; 0.5% sodium deoxycholate; 0.1% SDS; and 50 mm NaF) supplemented with Protease Inhibitor Cocktails (cOmplete, Mini, EDTA‐free, Sigma) and sodium orthovanadate as phosphatase inhibitor. The protein concentrations were estimated by Bradford protein assay (Bio‐Rad, Hercules, CA, USA), and the samples were treated with Laemmli sample loading buffer (Bio‐Rad), boiled, and subjected to SDS/PAGE and electrotransferred to polyvinylidene fluoride membranes. The membranes were blocked with 10% nonfat dry milk in PBS and 0.1% Tween‐20 (PBS‐T buffer) and incubated overnight at 4°C using the following primary antibodies: caspase‐3 (1 : 1000), PARP1 (1 : 1000) and GAPDH (1 : 10000) from Cell Signaling Technology, p53 (1 : 1000, cat. no. P53‐CM5P‐L) from Leica and mst1r polyclonal antibody (1 : 1000, cat. no. PA5‐71878) from Life Technologies, followed by horseradish peroxidase‐conjugated secondary antibodies (Bio‐Rad). Immunoblots were developed using enhanced chemiluminescence (ECL) reagents (Bio‐Rad) and imaged using a UVITEC imaging system. The original full scans are provided in Fig. S6, Fig. S7.

### 2.4 Chromatin immunoprecipitation (ChIP)

ChIP assay was performed using 1% formaldehyde for 10 min to cross‐link the proteins to the DNA, except for BRG1 ChIP where the cross‐linking was carried out for 30 min. The reaction was quenched with 0.125 m glycine. Then, the immunoprecipitation was conducted using Dynabeads Protein G (Invitrogen, cat. no. 10004D). The cross‐link was then reversed with Proteinase K (20 mg·mL^−1^, Thermo Scientific, Waltham, MA, USA) and 0.1 mg·mL^−1^ RNase A (Thermo Scientific). The DNA was purified by QIAquick PCR Kit (Qiagen) and measured using real‐time quantitative PCR. The following antibodies were used: anti‐p53 (Leica, cat. no. P53‐CM5P‐L), anti‐H2A.Z (Millipore, ABE1348), anti‐BRG1 (Abcam, ab110641) and mouse/rabbit IgG isotype control (Invitrogen, cat. no. 10400C/10500C).

### 2.5 RNA extraction, reverse transcription and real‐time qPCR analysis

RNA was extracted using RNeasy Mini Kit (Qiagen) according to the manufacturers’ protocols and reverse‐transcribed into cDNA utilizing SensiFAST cDNA Synthesis Kit (Meridian Bioscience, BIO‐65054). RT‐qPCR was performed with Fast SYBR Green PCR Master Mix (Applied Biosystems) and Quant Studio 5 System (Applied Biosystems). To calculate the relative RNA expression levels, TBP mRNA was used as a normalizer. The primers used for real‐time qPCR are reported in Table S1.

### 2.6 Live‐cell imaging analysis

Caspase activation was evaluated using IncuCyte® Live‐Cell Analysis Systems. The cells were transfected with siRNA and, 24 h later, seeded into a 96‐well plate. The day after, gemcitabine (at the specific contraction indicated in the figure legends) and IncuCyte® Caspase‐3/7 Green Dye for Apoptosis (Sartorius, cat. no. 4440) at 2,5 uM were added. The cells were placed into the IncuCyte® Live‐Cell Analysis System to monitor apoptosis every 3 h. The analysis was effectuated with incucyte Basic Analysis Software. The system generates a graph calculating the green area normalized by division with the phase area confluence.

### 2.7 Bioinformatic analyses

The data used for the Kaplan–Meier survival analysis were acquired from PanCancer Atlas, The Cancer Genome Atlas (TCGA) data set study. The whole patient cohort was divided into two groups based on the p53 status (mutated or not mutated). Instead of the pie plot analysis, the data were collected from different cBioPortal Pancreatic data set studies (TCGA, PanCancer Atlas; UTSW, Nature Commun 2015; QCMG, Nature 2016; ICGC, Nature 2012), the genetic alterations were extrapolated, and the pie plot was realized. Then, the mst1r mRNA expression was correlated with p53 status of PDAC patients through cBioPortal (https://www.cbioportal.org/). For the overall survival of MST1r expression, the patient cohort was divided into patients with higher and lower mst1r mRNA levels (based on the median cut‐off).

From ChIP‐Atlas (http://chip‐ atlas.org/peak_browser), Chip‐seq data were examined and Integrative Genomics Viewer (http://www.broadinstitute.org/igv/) was used to visualize peaks. Through obtained data on mst1r gene, the primers for ChIP assay were designed (listed in Table S1), based on peaks binding for NFYA (Chip‐seq id = SRX032491), Srebf1 (Chip‐seq id = SRX1650053), Ets2 (Chip‐seq id = SRX3730274) and trp63 (Chip‐seq id = SRX3205488), using UCSC Genome Browser (http://genome.ucsc.edu/index.html).

### 2.8 Cell cycle analysis

After transfections, cells were treated with gemcitabine at 10 and 100 nm concentrations. The cells were fixed in an equal volume of PBS 1x and methanol/acetone (4 : 1 (v/v) solution) overnight at 4 °C and incubated with 13 kunitz units solution of RNase (DNase free) at 37 °C for 15 min. Finally, cells were labelled with 100 mg·mL^−1^ of propidium iodide (P4170, Sigma) overnight at 4 °C in the dark. After one wash, cells were acquired using a CytoFLEX LX (Beckman Coulter, Brea, CA, USA) and analysed by cytexpert software (Beckman Coulter).

### 2.9 Statistics

The experiments were analysed using graphpad Prism 9.0 (graphpad Software Inc.). All results are expressed as the mean ± SEM or SD. RT‐qPCR data were analysed by the *t*‐test (*P < 0.05, **P < 0.01 and ***P < 0.001). For ATAC‐seq and RNA‐seq, rawLog2FC, shrunkenLog2FC and adjusted *P*‐value were used as statistical analysis.

The Mantel–Cox test was applied to the Kaplan–Meier approach to determine the significance of progress‐free survival and overall survival between different patients.

All the experiments were performed with at least two biological repeats.

## 3 Results and discussion

### 3.1 p53 mutations confer drug tolerance to standard chemotherapy in pancreatic adenocarcinoma

**Fig. 1:**
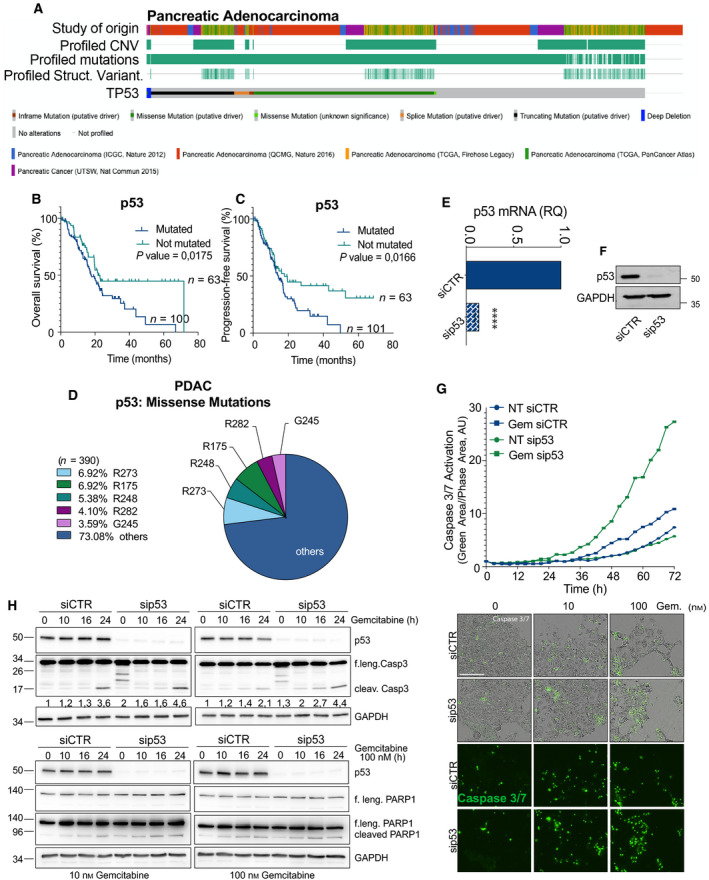
p53 mutants confer drug tolerance to pancreatic cancer. (A) Oncoprint plot reports *TP53* status in a meta‐analysis of 5 PDAC patient data sets (including QCMG, TCGA and ICGC). *n* = 1034. Source cbioportal.org. (B,C) Overall (B) and progression‐free (C) survival of p53 mutated/not mutated cohorts in the PDAC group of the TCGA PanCancer. *n* = 165. (D) Distribution of the most frequent TP53 missense mutations in the PDAC cohorts of the TCGA. (E,F) mRNA and protein level of p53^R270H^ following silencing in KPC^270^ pancreatic cancer cells (SEM; *t*‐test, *P*‐value < 0.0001). (G) *In vivo* live‐cell imaging analysis by IncuCyte platform to measure cell growth (phase contrast) and caspase‐3/7 activation (fluorescent green area) in KPCR^270^ cells following p53^R270H^ silencing and gemcitabine (Gem.) treatment. Microscopy images are representative of 72‐h time point. Scale bar indicates 200 microns. (H) Western blot analysis for PARP1 and caspase‐3 cleavage following p53^R270H^ silencing and gemcitabine treatment in KPC^270^ cells. The illustrated data in e‐h are representative of three biological repeats.

p53 is highly mutated across the largest majority of human cancers, and this often correlates with unfavourable prognosis and therapy resistance. PDAC is within the tumours with the highest rate of TP53 mutations, exceeding the overall average threshold of 50% of the cases (Fig. 1A). Analysis of PDAC patients from the TCGA PanCancer Atlas confirmed that TP53‐mutated status correlates with unfavourable prognosis (Fig. 1B), but more importantly indicated that dysfunctional TP53 alleles correlate with a significantly lower probability of progression‐free survival (PFS) (Fig. 1C). As PFS is a measurement of treatment effectiveness (NCI definition), this is suggestive of a predictive role for TP53 inactivation in the therapeutic response of PDAC patients.

80% of the TP53‐inactivating events is observed as missense mutations underlying expression of neomorphic gain‐of‐function mutants [3]; more specifically, the largest majority of mutations generally impinge on arginine 273 (R273) or 175 (R175) and 248 (R248) (Fig. 1D, Fig. S1A). Hence, we selected pancreatic adenocarcinoma cell lines derived from *pdx1‐CR*E mouse models with pancreas‐specific expression of oncogenic KRAS (*LSL‐KRAS^G12D^*) and p53^R270H^ mutation (homologue of human R273H) or p53^R172H^ mutation (homologue of human R175H), hereafter referred to as KPC^R270H^ or KPC^R172H^ cells. In agreement with our clinical analysis, p53^R270H^ conferred resistance to standard gemcitabine treatment in KPC^R270H^ cells. A modest time‐dependent increase in apoptotic caspase activation (caspase‐3/7) was observed in gemcitabine‐treated KPC^R270H^ cells, which was significantly enhanced upon p53^R270H^ silencing (Fig. 1E‐G, Fig S1B‐D). A similar trend was observed in the mouse KPC^R172H^ cells (carrying p53^R172H^) and the human PANC1 (carrying p53^R273H^) and HPAF II (carrying p53^P151S^) cell lines (Fig. S1E,G). Consistently with the *in vivo* live‐cell imaging results, western blot analyses confirmed enhanced apoptosis in p53^R270H^‐depleted KPC cells following gemcitabine treatment, as assessed by caspase‐3 and PARP1 cleavage (Fig. 1H). Gemcitabine alone or as a basic agent in combination with Abraxane (nab‐paclitaxel) is a first‐line treatment for PDAC patients, although often therapy resistance emerges leading to fatal outcome [16]. Hence, our data indicate that KPC^R270H^ cells display a p53^R270H^‐dependent drug‐tolerant phenotype that experimentally recapitulates *in vitro* the clinical scenario.

### 3.2 p53 mutant finely tunes chromatin landscape to dictate a therapy resistance transcriptional programme to pancreatic cancer cells

**Fig. 2:**
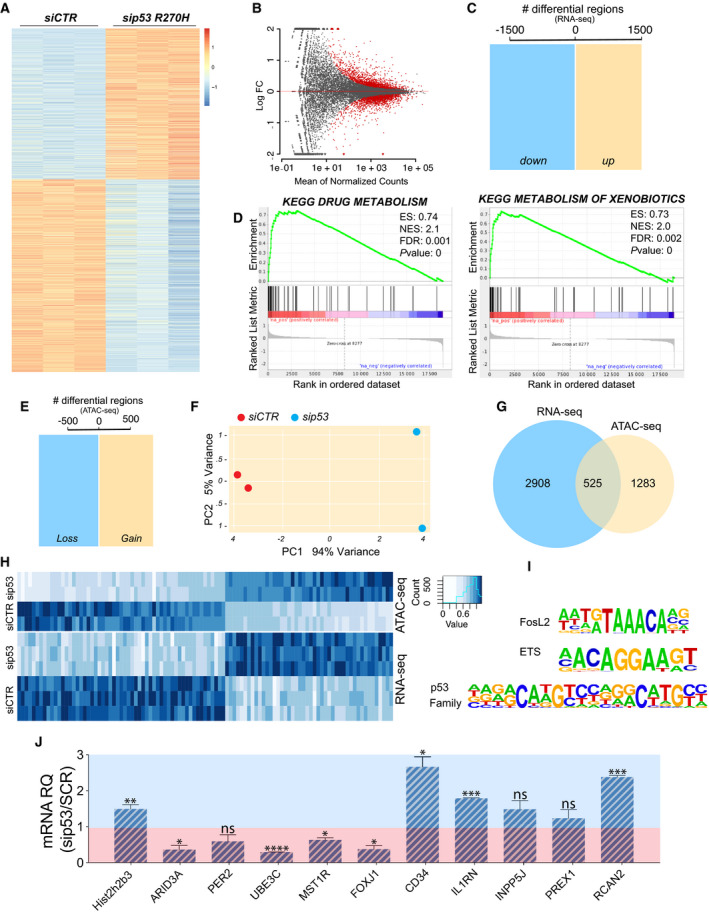
p53^R270H^ regulates transcriptional programme by finely tuning chromatin accessibility. (A‐C) RNA‐seq (*n* = 3) following p53^R270H^ silencing in KPC^R270H^ cells identified approximately 3000 differentially expressed genes. (D) Gene set enrichment analysis indicated ‘Drug Metabolism’ and ‘Metabolism of xenobiotics’ among the top enriched pathways. (E,F) ATAC‐seq (*n* = 2) identified more than 1700 genomic sites, whose accessibility is influenced by p53^R270H^ expression in KPC^R270H^ cells. (G,H) RNA‐seq and ATAC‐seq results display a significant overlap between genes differentially regulated and genomic sites whose accessibility is altered by p53^R270H^. (I) Motif analysis of the consensus sequences for transcriptional factors, which are known interactors of p53^R270H^. (J) RT‐qPCR analysis for the validation of the differentially expressed genes identified by RNA‐seq. The reported threshold divides the genes into two groups: p53^R270H^‐upregulated genes (red area) and p53^R270H^‐downregulated genes (blue area). Histograms report an average of three independent biological replicates +/− SD. The *t*‐test was used, and *P*‐values were calculated (not significant (ns) *P* ≥ 0.05, *P < 0.05, **P < 0.01, ***P < 0.001 and ****P < 0.0001.

GOF of p53 mutants has been for long postulated, while understanding of the underlying molecular mechanisms has not reached a very general consensus yet [2, 17]. To gain specific insights into the p53^R270H^‐dependent gemcitabine resistance of our KPC cell model, we performed an RNA‐seq‐based transcriptional profile following p53^R270H^ silencing. Approximately 3000 mRNA values were differentially expressed, with both the upregulated and downregulated groups equally represented and distributed (Fig. 2A–C, Fig. S2A,B and Table S1). In keeping with the therapy resistance phenotype, gene set enrichment analysis (GSEA) indicated ‘Drug Metabolism’ and ‘Metabolism of Xenobiotics’ among the top enriched pathways represented in the differentially expressed genes (Fig. 2D). Thus, a direct relationship between phenotype and transcriptional changes clearly emerged in our experimental model. We next corroborated the transcriptional profile with an assay for transposase‐accessible chromatin (ATAC)‐seq to determine the changes in the chromatin accessibility associated with p53^R270H^ expression. Approximately 1300 chromatin loci displayed altered chromatin accessibility upon p53^R270H^ silencing. Also in this case, gains and losses of accessibility were equally represented and distributed (Fig. 2E, Fig. S3 and Table S1). The p53^R270H^‐dependent remodelling of the chromatin landscape appeared to finely tune accessibility of site‐specific loci (Fig. 2F and Fig. S2). Although this is the first p53 mutant‐dependent ATAC‐seq profiling, these data are in full agreement with the previous reports describing site‐specific capability of p53 R273H/R270H to modulate the activity of SWI/SNF chromatin remodelling complex [8, 18] and COMPASS complex [19] and influence chromatin conformation.

We next integrated the RNA‐seq with the ATAC‐seq results and identified approximately 500 chromatin sites, where an alteration in the accessibility was compatible with changes in the expression of genes in the close proximity (Fig. 2G,H). Interestingly, consensus motif analysis identified binding sequences for transcriptional factors that are known p53 mutant interactors, such as ETS, p53 family members [17] (Fig. 2I). Hence, our data indicate that at least in part mutant p53 GOF seems to co‐opt site‐specific chromatin regulations to alter gene expression and cellular phenotype. A selection of genes, whose expression resulted altered by p53^R270H^‐dependent chromatin regulation, was tested by RT‐qPCR to validate the RNA‐seq data; some of these appeared to be possibly involved in the PDAC chemoresistance phenotype (Fig. 2J).

### 3.3 p53 mutants regulate MST1r expression conferring gemcitabine resistance to pancreatic cancer cells

**Fig. 3:**
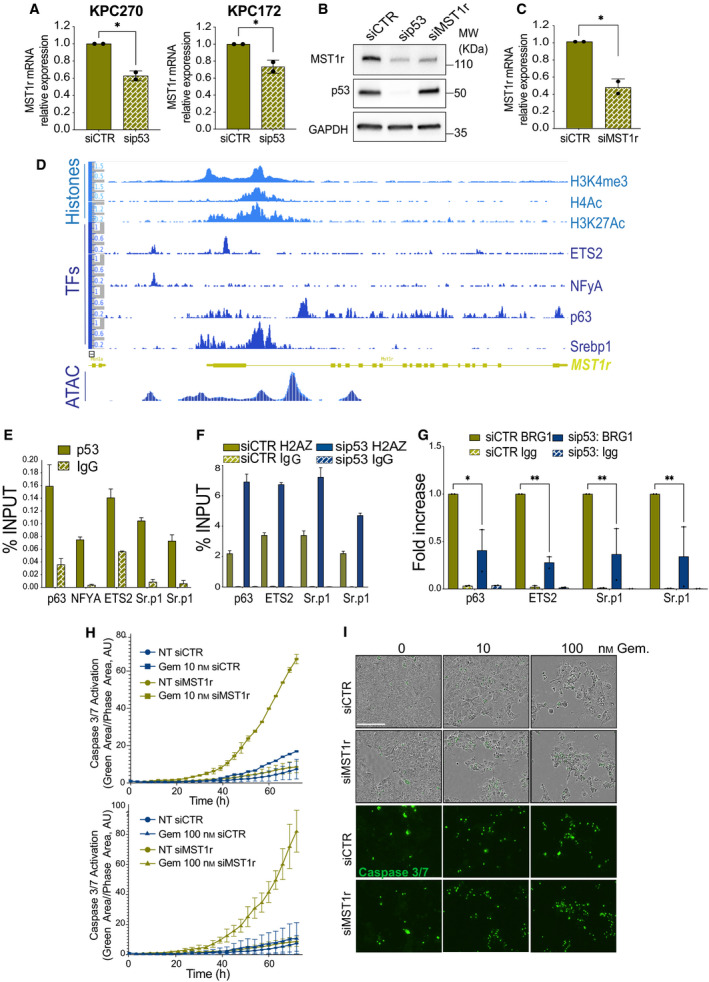
p53^R270H^/MST1r axis confers drug tolerance to pancreatic cancer. (A‐C) RT‐qPCR (*n* = 2) and western blot display mRNA or protein level of MST1r following p53^R270H^ or p53^R172H^ silencing in KPC cells. Bars indicate SD. Experiments were repeated at least three times. A *t*‐test was calculated (*P*‐value < 0.05). (D) ChIP‐seq profile of histone post‐translational modifications, transcriptional factor (TF) binding and ATAC‐seq in the genomic region of mouse MST1r. (E‐G) ChIP‐qPCRs report p53^R270H^ binding (E), H2AZ content (F) or Brg1 binding (G) in the region corresponding to p63, ETS2 and Srepb1 (Sr.p1) binding (from panel (D) in KPC^R270H^‐untreated cells (E) or following p53^R270H^ silencing (F and G). E and F report representative replicates of at least three independent biological replicates (bars indicate SD of technical replicates), G reports an average of fold over control of two independent biological replicates (bars indicate SE of biological replicates), two‐way ANOVA test was used, *P*‐values: * *P* < 0,05 and ** *P* < 0.01. (H,I) *In vivo* live‐cell imaging analysis by IncuCyte platform measured cell growth (phase contrast) and caspase‐3/7 activation (fluorescent green area) in KPC^R270H^ cells following MST1r silencing and gemcitabine (Gem) treatment. Scale bar indicates 200 microns. The data show a representative experiment of two biological replicates.

The expression of p53^R270H^ dictates a specific gene expression programme (Fig. 2), which include many candidate genes for the regulation of gemcitabine resistance. Among these, the macrophage‐stimulating protein 1 receptor, MST1r, resulted was positively regulated by both p53^R270H^ and p53^R172H^, as p53 silencing appeared to affect its expression level in KPC^R270H^ and KPC^R172H^ cells (Fig. 3A).

MST1r is a candidate target for novel anticancer therapeutic approaches; it functions as a tyrosine kinase receptor of the macrophage‐stimulating protein 1 (MSP1), and MSP1‐MST1r signalling has been proven to control survival, invasion and chemoresistance of many cancer types. A fraction of cancers, including PDAC, display mutations in MST1r gene; alternatively, upregulation of MST1r can underlie hyperactivation of this receptor in cancer cells, leading to protumorigenic phenotype [20]. Consistently, overexpression or deletion of MST1r in KRAS‐driven mouse pancreatic cancer model affects the development and progression of PDAC phenotype, influencing the immune infiltrate [21]. Although MST1r has attracted increasing attention in the past decade, molecular bases for its pathological upregulation are currently unknown.

MST1r mRNA level was downregulated upon p53 silencing (Fig. 3A); this downregulation substantially affected the protein level (Fig. 3B,C). Overexpression of MST1r in PDAC mouse models was associated with upregulation of its ligand [21]; we therefore also measured the level of MSP1, whose expression did not appear, however, to be significantly influenced by p53^R270H^ (Fig. S4A) nor substantially expressed at the background (Ct > 31). Hence, this suggested that the MST1r expression is the main mechanism of regulation that p53 mutant mediates on this signalling.

Analysis of ChIP‐seq data indicated the presence of multiple binding sites between promoter and intron 1 of MST1r gene locus for transcriptional factors interacting with mutant p53 (Fig. 3D), such as p63, ETS2, NY‐F and Srebp (reviewed in Pilley et al. [3]). The area appeared rich in permissive post‐translational histone marks, such as H3K4me3 (trimethylations of lysine 4 of histone 3), H3K27Ac (acetylation of lysine 27 of histone 3) and H4Ac (acetylation of histone 4), confirming the regulatory nature of these genomic regions (Fig. 3D). Remarkably, the changes in the chromatin accessibility profile following p53^R270H^ silencing (ATAC‐seq) were highly aligned to the position of p63, NF‐Y, ETS2 and Srebp binding picks (Fig. 3D), thus suggesting a causative relationship for p53^R270H^ binding to these sites and the chromatin conformation. Hence, to experimentally prove this relationship we first assessed p53^R270H^ binding by ChIP on endogenous p53 mutant in KPC^R270H^ cells. qPCR detected the presence of p53^R270H^ on the area corresponding to p63, NF‐Y, ETS 2 and Srebp binding (Fig. 3E); specificity of p53 ChIP was confirmed by lack of p53 enrichment in p53^‐\‐^ cells, KP^fl^C (Fig. S4B). Next, we validated the ability of p53^R270H^ to alter chromatin conformation by assessing the content of the H2 isoform H2AZ as a marker of different nucleosome positioning. H2AZ ChIP indicated substantial changes to chromatin organization in the areas where p63, NF‐Y, ETS 2 and Srebp binding was expected (Fig. 3F). In addition, the binding of Brg1, an ATPase subunit of the SWI/SNF chromatin remodelling complex, to these specific loci appeared to be influenced by p53^R270H^ expression (Fig. 3G), consistent with the previously reported implication of SWI/SNF in p53 mutant GOF effect [8, 18]. Overall, our data demonstrate that p53^R270H^ regulates MST1r, binds its gene regulatory regions and alters the chromatin landscape. These data validated the results of our ATAC‐seq and RNA‐seq profiles, highlighting the contribution of the epigenetic regulations as underlying mechanisms of p53 mutant GOF.

Altered response to drugs appeared as a signature of p53^R270H^‐dependent transcriptome (Fig. 2); consistently, p53 mutants affect drug response *in vitro* and in patients (Fig. 1). Hence, we investigated the effect of MST1r expression on the KPC^R270H^ cell response to gemcitabine. Silencing of MST1r substantially affected the response of KPC cells to gemcitabine, leading to a massive increase in caspase‐3/7 activation in live‐cell imaging experiments (Fig. 3H,I). This result strongly recapitulated the p53^R270H^ effect on KPC cell response to gemcitabine, thus suggesting that the p53^R270H^/MST1r axis is representative of the molecular underlying mechanisms of the p53^R270H^‐dependent chromatin landscape/transcriptome responsible for the drug‐tolerant phenotype.

### 3.4 MST1r expression correlates with p53 status and pancreatic cancer prognosis

**Fig. 4:**
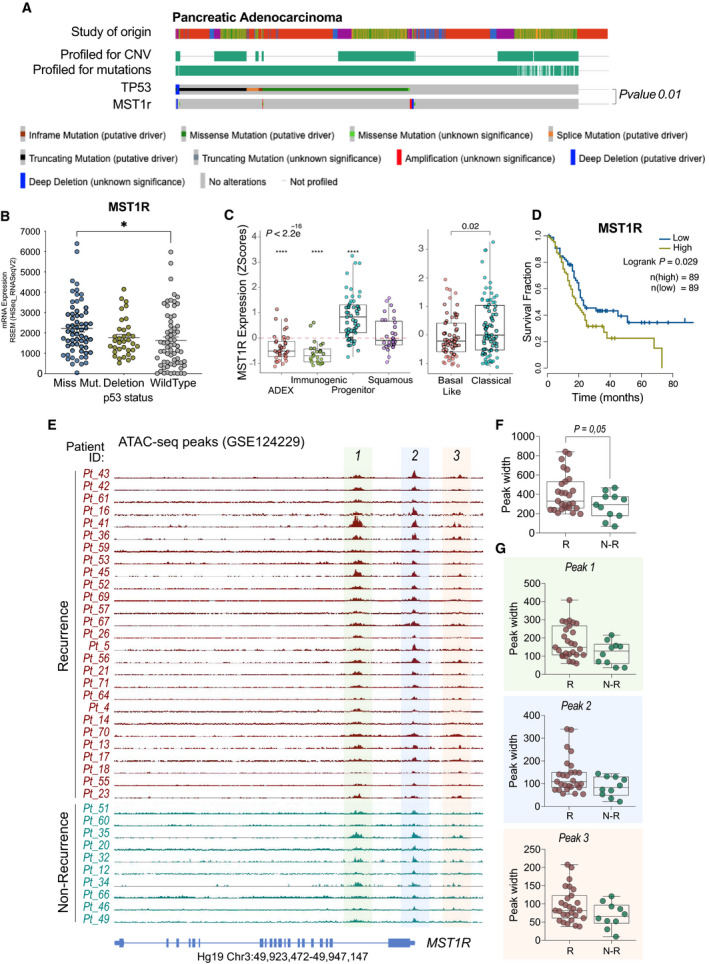
MST1r has a prognostic value and correlates with p53 status in human pancreatic cancer. (A) Oncoprint plot reports *TP53* and MST1r mutational status in a meta‐analysis of 5 PDAC patient data sets (including QCMG, TCGA and ICGC). *N* = 960. Source cbioportal.org. (B) Expression level of MST1r in p53 mutant (missense or deletion) and p53 wt cohorts of PDAC patients from TCGA data set. (C) Expression level of MST1r in different subtypes of PDAC. (D) Overall survival of MST1r high/low cohorts of patients from TCGA data set. (E) Chromatin accessibility on MST1r genomic site (ATAC‐seq) of PDAC patients (GSE124229) stratified for the recurrence status. (F,G) Quantification of peak width in PDAC patients with recurrence (R) and no recurrence (N‐R). All peaks are reported in (F); peaks 1, 2 and 3 are reported individually in (G). The illustrated data (B, C, F and G) report a dot for each individual patient ± SEM. The *P*‐value was calculated using the one‐way ANOVA test (B,C) (*P < 0.05 and ****P < 0.0001) or the *t*‐test (F,G).

To verify whether the p53^R270H^/MST1r axis identified in an experimental model of pancreatic cancer was conserved in human cancers, we analysed a clinical cohort of PDAC patients including genomic and transcriptomic data. Firstly, we detected genetic alterations in MST1r genes in a small fraction of the patients. Remarkably, although p53 mutation was highly represented in the cohort, mutually exclusivity emerged with MST1r genetic alterations (Fig. 4A), indicating that p53 mutations relieve selective pressure for MST1r mutations, in agreement with our data that support a mechanism for enhanced gene transcription of MST1r when p53 is mutated. The relationship between MST1r gene expression and p53 mutant was further proved by the higher level of MST1r mRNA in the cohort of patients carrying mutations in p53 compared with the p53 wild‐type (wt) cohort (Fig. 4B). A similar trend was confirmed for a subgroup of genes from the validation of our RNA‐seq analysis in Fig. 2J (Fig. S5). Moreover, MST1r high expression seemed to define specific subtypes of PDAC, such as ‘progenitor’ and ‘classical’ (Fig. 4C) and expression of MST1r stratified the patients in two well‐defined cohorts with different prognoses. In keeping with the suggested role in drug tolerance, a high level of MST1r correlates with a significantly lower survival expectation in PDAC patients (Fig. 4D). Finally, we correlated accessibility of MST1r chromatin locus with PDAC recurrence analysing chromatin accessibility profiles in a cohort of surgically resected PDAC (ATAC‐seq data set GSE124229 [22]). Wider chromatin accessibility of MST1r locus was observed in samples from patients whose disease relapsed postsurgery (Fig. 4E–G), confirming our data regarding the importance of this epigenetic regulation in human disease settings. Hence, this latter set of data supports clinical relevance for MST1r in pancreatic cancer progression and supports the relevance of p53^R270H^/MST1r axis. Overall, our data indicate that a p53 mutant‐mediated mechanism of chromatin remodelling leads to deregulation of gene expression, including genes such as MST1r, that plays substantial role in therapy resistance and PDAC prognosis.

## 4 Conclusions

The mechanisms underlying p53 mutant GOF are still largely debated, although recent evidence indicates a direct contribution of epigenetic and chromatin regulations [2]. Our data report the first p53 mutant‐dependent chromatin accessibility profile and suggest an alteration in chromatin state as a mechanism of p53 mutant GOF. Importantly, p53^R270H^ appears to finely tune chromatin accessibility at specific chromatin loci, and in a substantial number of cases, these correlate with alteration in gene expression. In keeping with this, previous studies have reported functional and biochemical interactions between p53 mutant and chromatin‐modifying complexes, such as SWI/SNF and COMPASS [8, 18, 19]. Thus, here we formally assess the capability of p53 mutant GOF to function through a mechanism of regulation of chromatin landscape. However, a significant part of the genes differentially expressed in p53^R270H^‐depleted cells does not correlate with a change in chromatin accessibility in the corresponding gene locus; hence, a substantial contribution of p53 mutant GOF effects must be mediated by alternative mechanisms.

A drug‐tolerant phenotype is promptly acquired by the largest majority of PDAC patients. Remarkably, while the progression of PDAC in the first stages is clearly associated with genomic evolution, pronounced cellular plasticity of PDAC phenotype in later stages (i.e. metastasis and drug resistance) seems predominantly associated with epigenetic mechanisms [23, 24]. This makes the switch of phenotypic states possibly reversible, opening to epigenetic treatment strategies [15, 25]. The epigenetic‐dependent activation of p53^R270H^/MST1r axis and the consequent drug‐tolerant phenotype fall into this possible scenario. Hence, identification of the basis for PDAC epigenetic deregulation and more in particular the dissection of the interaction between genetic events, such as p53 mutations, and epigenetic deregulation should be given emphasis for the identification of therapeutic strategies to cure standard therapy‐refractory PDAC.

## Conflict of interest

The authors declare no conflict of interest.

## Data accessibility

Data are available upon request, and relevant data sets will be deposited in public repositories.

## Author contributions

CZ conducted research and analysed the data. EP helped with data analysis. VC, AM and GM provided reagents and analytical tools. IA conceived the research and revised the manuscript. IA and CZ wrote the manuscript. All the authors approved the final version of the manuscript.

## Supporting information


**Fig. S1**. P53 missense mutations lend resistance to treatment in pancreatic cancer.

**Fig. S2**. Transcriptional signature variations after p53^R270H^ deletion.

**Fig. S3**. Modification in chromatin accessibility due to p53^R270H^.

**Fig. S4**. P53 mutant‐dependent variation of MST1 mRNA expression and control ChIP in p53 null cell line.

**Fig. S5**. Expression level of RNA‐seq‐ identified genes in PDAC patients.

**Fig. S6**. Full membrane images of the western blot data reported in main figures.

**Fig. S7**. Full membrane images of the western blot data reported in main figures.

**Table S1**. Q‐PCR primers.

## References

[1] Kastenhuber ER , Lowe SW . Putting p53 in context. Cell. 2017;170:1062–78. 28886379 10.1016/j.cell.2017.08.028 PMC5743327

[2] Amelio I , Melino G . Context is everything: extrinsic signalling and gain‐of‐function p53 mutants. Cell Death Discov. 2020;6:16. 32218993 10.1038/s41420-020-0251-x PMC7090043

[3] Pilley S , Rodriguez TA , Vousden KH . Mutant p53 in cell‐cell interactions. Genes Dev. 2021;35:433–48. 33861719 10.1101/gad.347542.120 PMC8015724

[4] Celardo I , Melino G , Amelio I . Commensal microbes and p53 in cancer progression. Biol Direct. 2020;15:25. 33213502 10.1186/s13062-020-00281-4 PMC7678320

[5] Kadosh E , Snir‐Alkalay I , Venkatachalam A , May S , Lasry A , Elyada E , et al. The gut microbiome switches mutant p53 from tumour‐suppressive to oncogenic. Nature. 2020;586:133–8. 32728212 10.1038/s41586-020-2541-0 PMC7116712

[6] Muller PA , Caswell PT , Doyle B , Iwanicki MP , Tan EH , Karim S , et al. Mutant p53 drives invasion by promoting integrin recycling. Cell. 2009;139:1327–41. 20064378 10.1016/j.cell.2009.11.026

[7] Weissmueller S , Manchado E , Saborowski M , Morris JPT , Wagenblast E , Davis CA , et al. Mutant p53 drives pancreatic cancer metastasis through cell‐autonomous PDGF receptor beta signaling. Cell. 2014;157:382–94. 24725405 10.1016/j.cell.2014.01.066 PMC4001090

[8] Amelio I , Mancini M , Petrova V , Cairns RA , Vikhreva P , Nicolai S , et al. p53 mutants cooperate with HIF‐1 in transcriptional regulation of extracellular matrix components to promote tumor progression. Proc Natl Acad Sci USA. 2018;115:E10869–78. 30381462 10.1073/pnas.1808314115 PMC6243248

[9] Amelio I , Melino G . The p53 family and the hypoxia‐inducible factors (HIFs): determinants of cancer progression. Trends Biochem Sci. 2015;40:425–34. 26032560 10.1016/j.tibs.2015.04.007

[10] Moon SH , Huang CH , Houlihan SL , Regunath K , Freed‐Pastor WA , Morris JPT , et al. p53 Represses the mevalonate pathway to mediate tumor suppression. Cell. 2019;176(564–580):e519. 10.1016/j.cell.2018.11.011 PMC6483089 30580964

[11] Do PM , Varanasi L , Fan S , Li C , Kubacka I , Newman V , et al. Mutant p53 cooperates with ETS2 to promote etoposide resistance. Genes Dev. 2012;26:830–45. 22508727 10.1101/gad.181685.111 PMC3337457

[12] Mizrahi JD , Surana R , Valle JW , Shroff RT . Pancreatic cancer. Lancet. 2020;395:2008–20. 32593337 10.1016/S0140-6736(20)30974-0

[13] Yachida S , Iacobuzio‐Donahue CA . Evolution and dynamics of pancreatic cancer progression. Oncogene. 2013;32:5253–60. 23416985 10.1038/onc.2013.29 PMC3823715

[14] Hruban RH , Adsay NV , Albores‐Saavedra J , Compton C , Garrett ES , Goodman SN , et al. Pancreatic intraepithelial neoplasia: a new nomenclature and classification system for pancreatic duct lesions. Am J Surg Pathol. 2001;25:579–86. 11342768 10.1097/00000478-200105000-00003

[15] Hessmann E , Johnsen SA , Siveke JT , Ellenrieder V . Epigenetic treatment of pancreatic cancer: is there a therapeutic perspective on the horizon? Gut. 2017;66:168–79. 27811314 10.1136/gutjnl-2016-312539 PMC5256386

[16] Weinberg BA , Yabar CS , Brody JR , Pishvaian MJ . Current standards and novel treatment options for metastatic pancreatic adenocarcinoma. Oncology. 2015;29(809–820):886. 26573060

[17] Pitolli C , Wang Y , Mancini M , Shi Y , Melino G , Amelio I . Do Mutations Turn p53 into an Oncogene? Int J Mol Sci. 2019;20:6241. 10.3390/ijms20246241 PMC6940991 31835684

[18] Pfister NT , Fomin V , Regunath K , Zhou JY , Zhou W , Silwal‐Pandit L , et al. Mutant p53 cooperates with the SWI/SNF chromatin remodeling complex to regulate VEGFR2 in breast cancer cells. Genes Dev. 2015;29:1298–315. 26080815 10.1101/gad.263202.115 PMC4495400

[19] Zhu J , Sammons MA , Donahue G , Dou Z , Vedadi M , Getlik M , et al. Gain‐of‐function p53 mutants co‐opt chromatin pathways to drive cancer growth. Nature. 2015;525:206–11. 26331536 10.1038/nature15251 PMC4568559

[20] Yao HP , Zhou YQ , Zhang R , Wang MH . MSP‐RON signalling in cancer: pathogenesis and therapeutic potential. Nat Rev Cancer. 2013;13:466–81. 23792360 10.1038/nrc3545

[21] Babicky ML , Harper MM , Chakedis J , Cazes A , Mose ES , Jaquish DV , et al. MST1R kinase accelerates pancreatic cancer progression via effects on both epithelial cells and macrophages. Oncogene. 2019;38:5599–611. 30967626 10.1038/s41388-019-0811-9 PMC6625868

[22] Dhara S , Chhangawala S , Chintalapudi H , Askan G , Aveson V , Massa AL , et al. Pancreatic cancer prognosis is predicted by an ATAC‐array technology for assessing chromatin accessibility. Nat Commun. 2021;12:3044. 34031415 10.1038/s41467-021-23237-2 PMC8144607

[23] Makohon‐Moore A , Iacobuzio‐Donahue CA . Pancreatic cancer biology and genetics from an evolutionary perspective. Nat Rev Cancer. 2016;16:553–65. 27444064 10.1038/nrc.2016.66 PMC5739515

[24] Reiter JG , Iacobuzio‐Donahue CA . Pancreatic cancer: pancreatic carcinogenesis ‐ several small steps or one giant leap? Nat Rev Gastroenterol Hepatol. 2016;14:7–8. 28003666 10.1038/nrgastro.2016.190

[25] Sharma SV , Lee DY , Li B , Quinlan MP , Takahashi F , Maheswaran S , et al. A chromatin‐mediated reversible drug‐tolerant state in cancer cell subpopulations. Cell. 2010;141:69–80. 20371346 10.1016/j.cell.2010.02.027 PMC2851638

