# Supplementary figures and images for: p53 mutations define the chromatin landscape to confer drug tolerance in pancreatic cancer
Source: Mol Oncol. 2022 Feb 11;16(6):1259–71. doi: 10.1002/1878-0261.13161 (PMC8936522; doi:10.1002/1878-0261.13161)

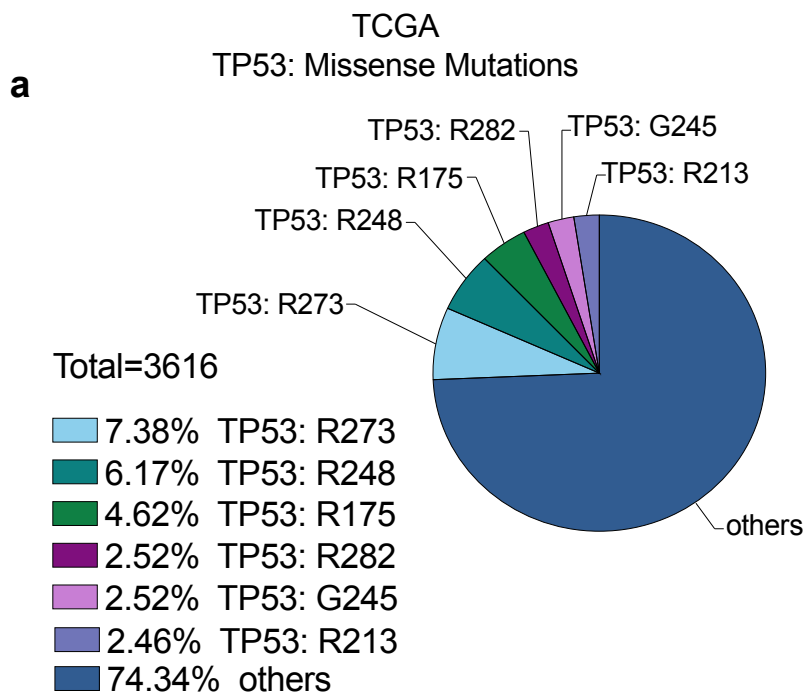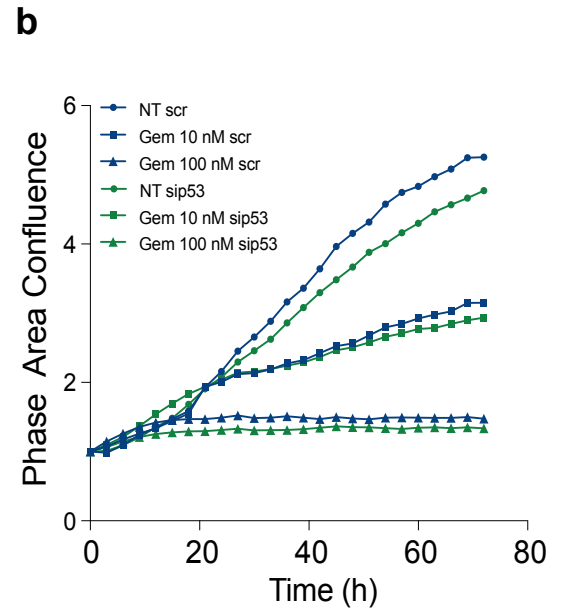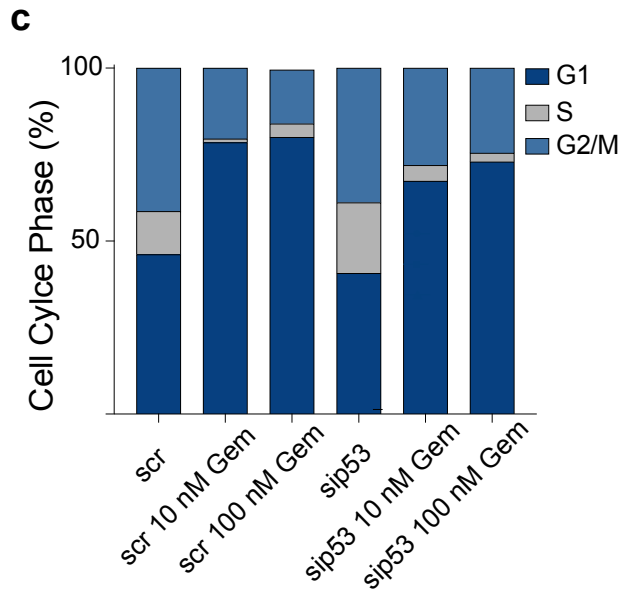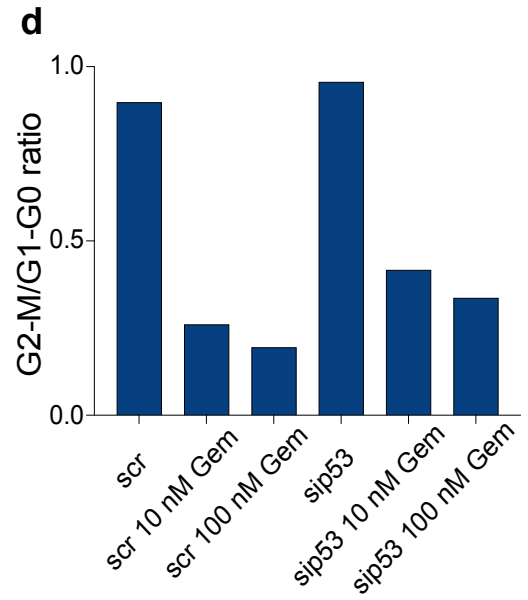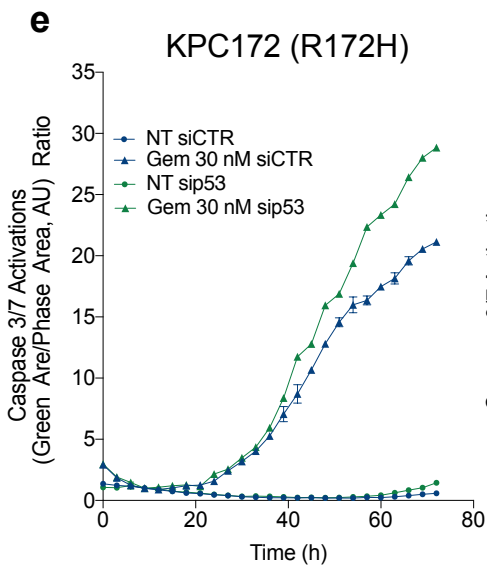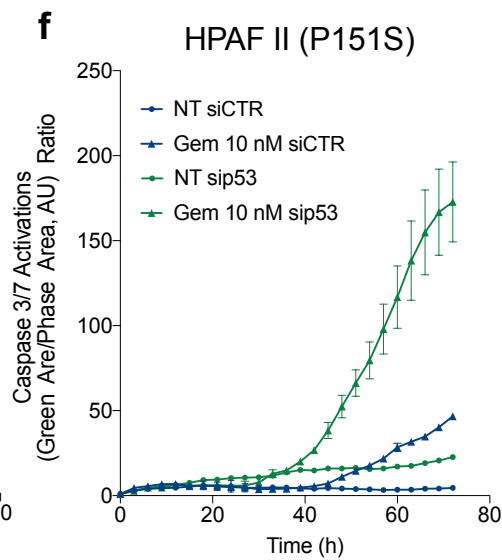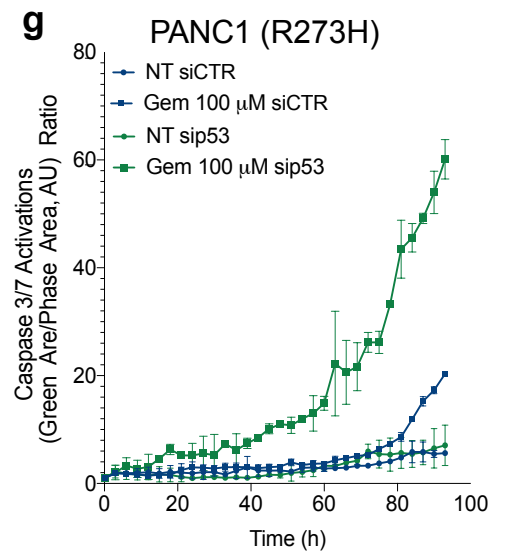

Supplement: Supplementary file 1 — Fig. S1. P53 missense mutations lend resistance to treatment in pancreatic cancer. [file MOL2-16-1259-s008.pdf]

**a**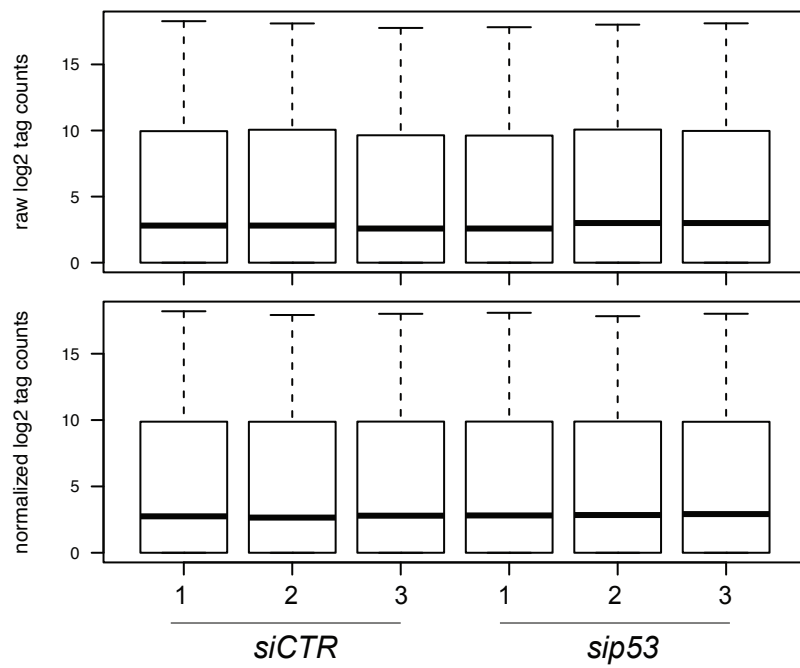**b**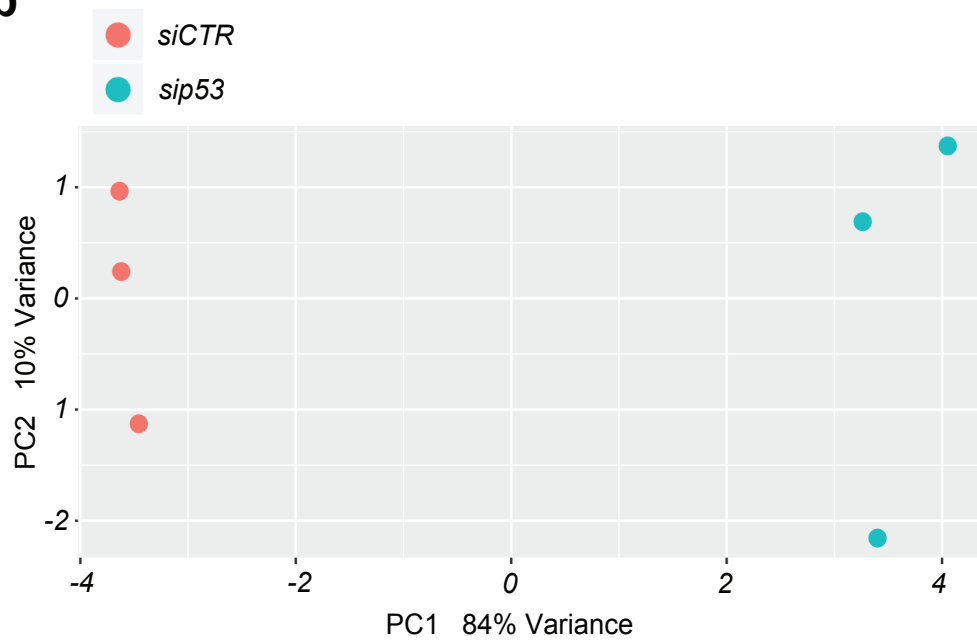

Supplement: Supplementary file 2 — Fig. S2. Transcriptional signature variations after p53R270H deletion. [file MOL2-16-1259-s002.pdf]

**a**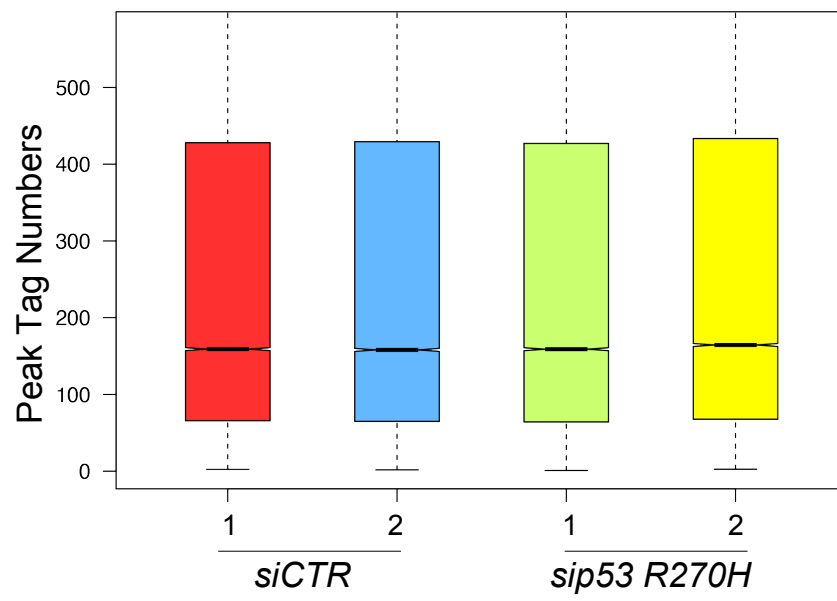**b**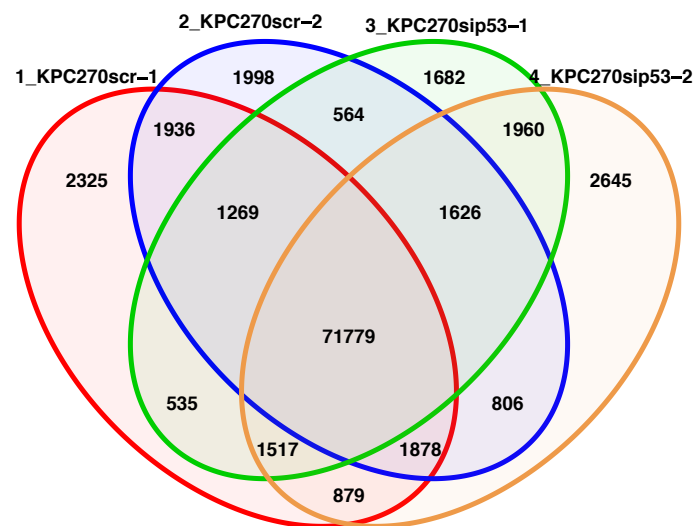

Supplement: Supplementary file 3 — Fig. S3. Modification in chromatin accessibility due to p53R270H. [file MOL2-16-1259-s004.pdf]

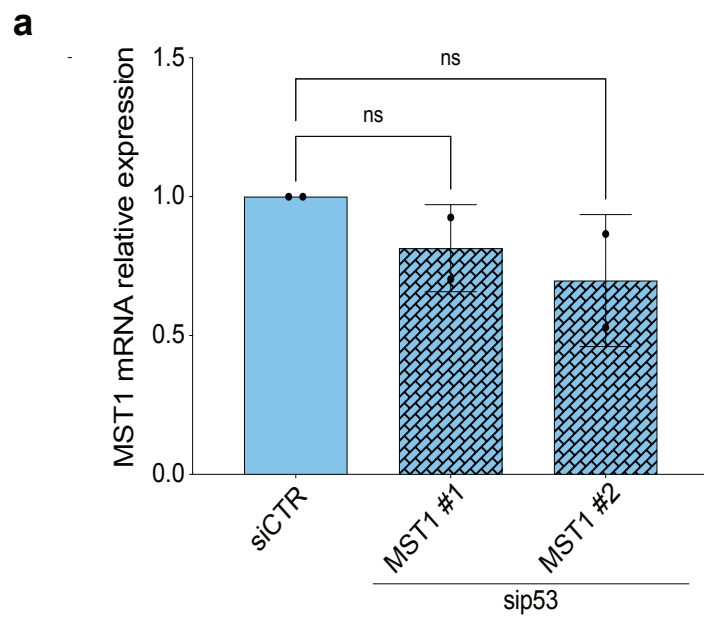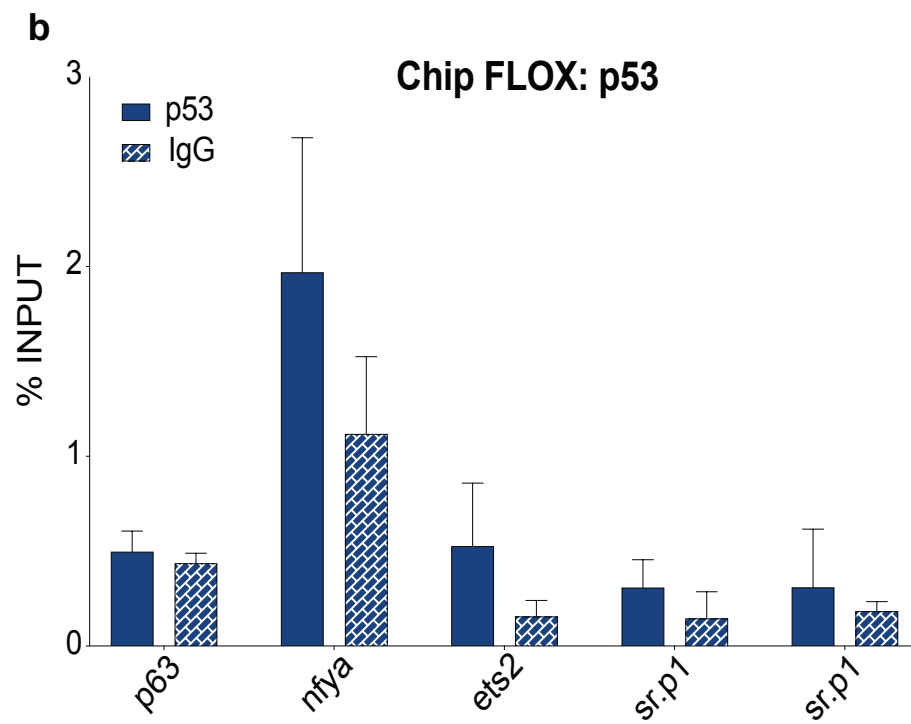

Supplement: Supplementary file 4 — Fig. S4. P53 mutant‐dependent variation of MST1 mRNA expression and control ChIP in p53 null cell line. [file MOL2-16-1259-s001.pdf]

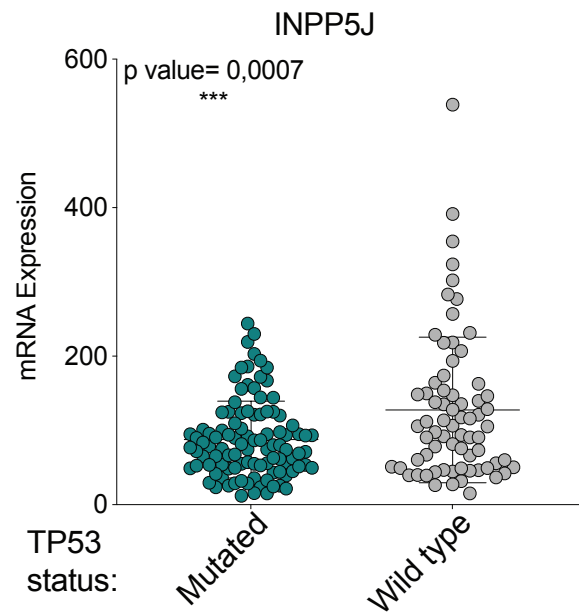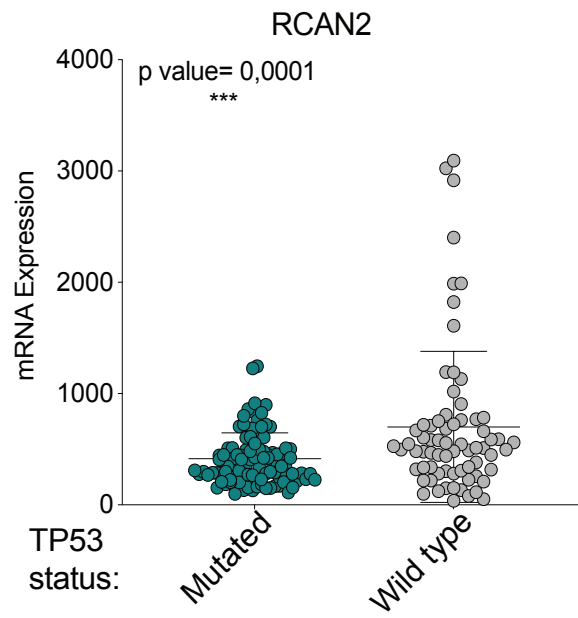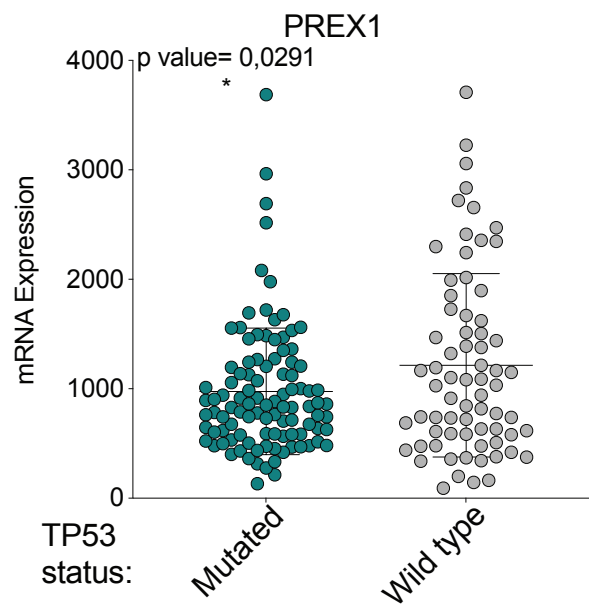

Supplement: Supplementary file 5 — Fig. S5. Expression level of RNA‐seq‐ identified genes in PDAC patients. [file MOL2-16-1259-s005.pdf]

**Figure 1 f**

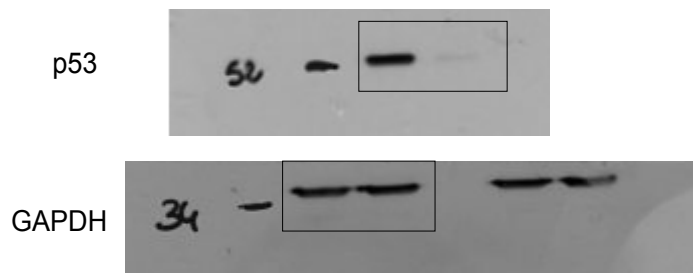

**Figure 1 h**

Gemcitabine  
10 nM (h)

Gemcitabine  
100 nM (h)

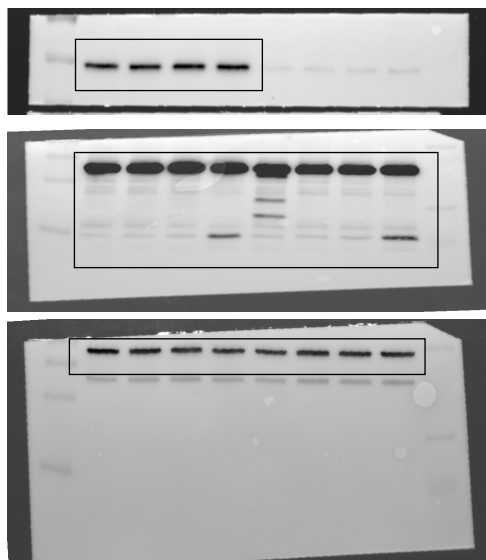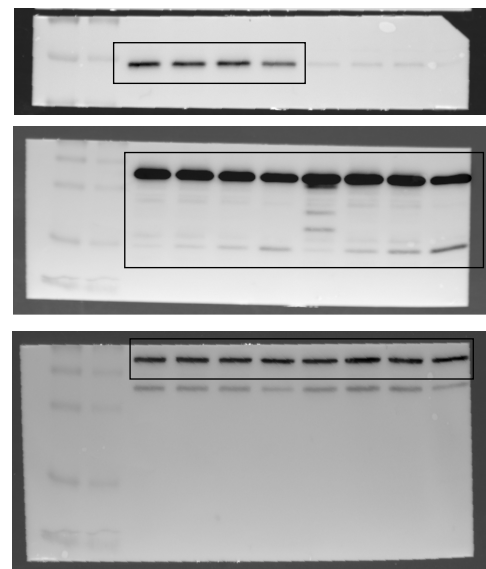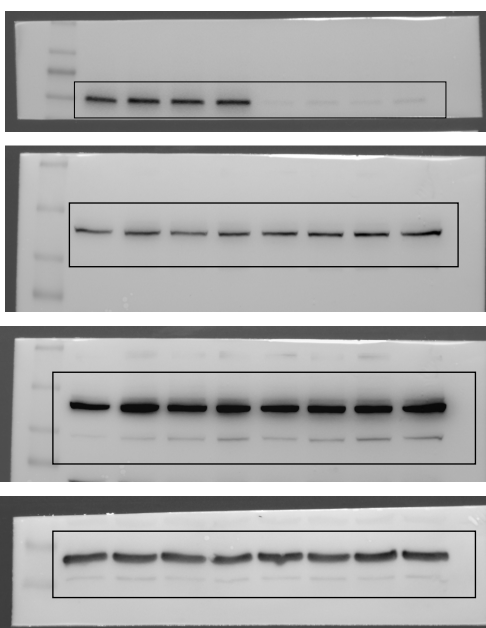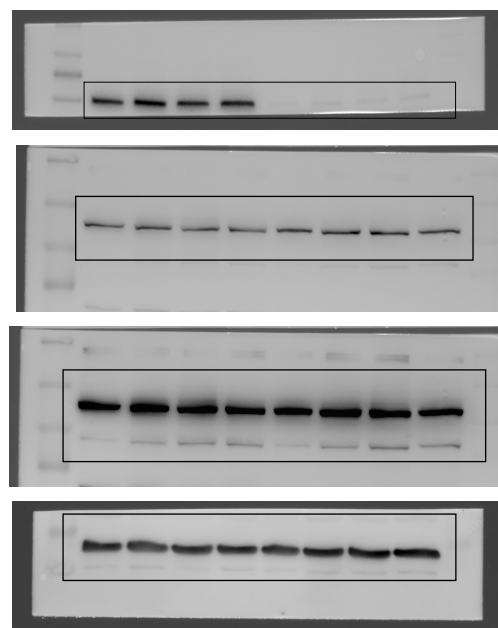

Supplement: Supplementary file 6 — Fig. S6. Full membrane images of the western blot data reported in main figures. [file MOL2-16-1259-s007.pdf]

**Figure 3 b**

MST1R

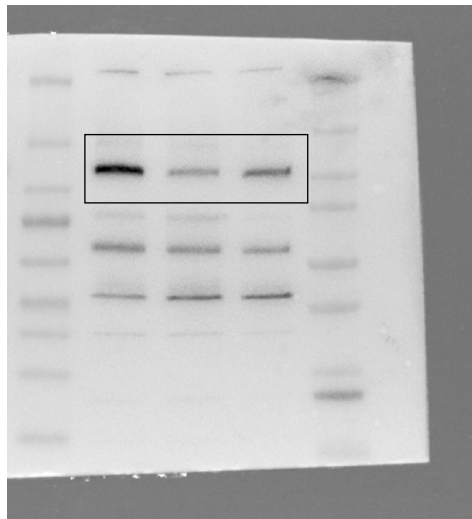

p53

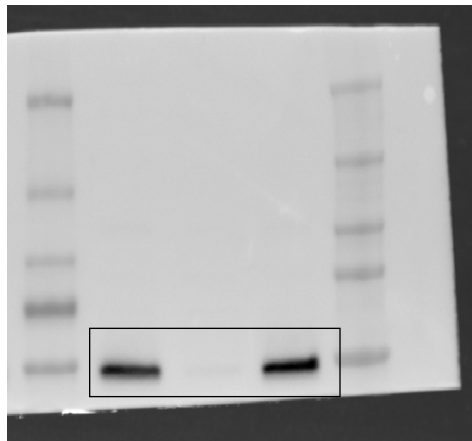

GAPDH

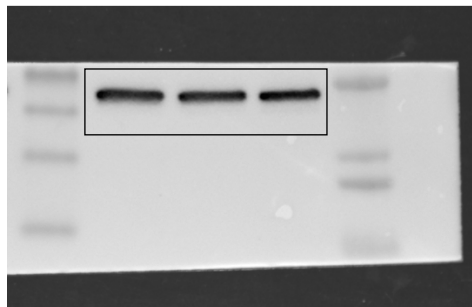

Supplement: Supplementary file 7 — Fig. S7. Full membrane images of the western blot data reported in main figures. [file MOL2-16-1259-s003.pdf]
