# Supplementary material for: p53 mutations define the chromatin landscape to confer drug tolerance in pancreatic cancer
Source: Mol Oncol. 2022 Feb 11;16(6):1259–71. doi: 10.1002/1878-0261.13161 (PMC8936522; doi:10.1002/1878-0261.13161)
Supplement: Supplementary file 8 — Table S1. Q‐PCR primers. [file MOL2-16-1259-s006.docx]

**p53 mutations define the chromatin landscape to confer drug tolerance in pancreatic cancer**

*Carlotta Zampieri^1^, Emanuele Panatta^1^, Vincenzo Corbo^2^, Alessandro Mauriello^1^, Gerry Melino^1^ and Ivano Amelio^1,3,*^*

^1^ Department of Experimental Medicine, TOR, University of Rome Tor Vergata, 00133 Rome, Italy.

^2^ University of Verona, Verona, Italy.

^3^ School of Life Sciences, University of Nottingham, Nottingham, UK.

* Email: ivano.amelio@uniroma2.it

**Supplementary Figure 1. p53 missense mutations lend resistance to treatment in pancreatic cancer. a)** Distribution of the most frequent TP53 missense mutations in the PanCancer dataset TCGA (n=3616) from cBioportal. **b)** *In vitro* live cell imaging analysis by IncuCyte platform to measure cell growth (phase contrast) in KPC^R270H^ cells following p53^R270H^ silencing and gemcitabine (Gem) treatment. **c,d**) Cell cycle analysis (c) and G2-M/G0-G1 ratio of KPC^R270H^ cells following p53^R270H^ silencing and gemcitabine treatment. **e-g**) In vivo live cell imaging analysis by IncuCyte platform to measure cell growth (phase contrast) and caspase 3/7 activation (fluorescent green area) in KPC^R172H^, HPAF II and PANC1 cells following p53 mutant silencing and gemcitabine treatment. The results display a representative experiment of two (c and d) or three (b,e-g) biological replicates.

**Supplementary Figure 2. Transcriptional signature variations after p53^R270H^ deletion. a)** Boxplot of tag counts shows the distribution of log2 fragment counts from the RNA-seq in genes for each sample before (top panel) and after (bottom panel) normalization. **b**) Principal component analysis for the RNA-seq data.

**Supplementary Figure 3. Modification in chromatin accessibility due to p53^R270H^. a)** Boxplot of tag counts show the distribution of log2 fragment counts from the ATAC-seq in genes for each sample before (top panel) and after (bottom panel) normalization. **b**) The overlap between peaks is reported in the VENN diagram. The diagram shows the number of Merged Regions for the various categories/overlap sets.

**Supplementary Figure 4. p53 mutant-dependent regulation of MST1 expression and control ChIP in p53 null cell line. a)** RT-qPCR assessing MST1 mRNA in KPC^R270H^ cells following p53 mutant silencing. ns: not significant (n=2). **b)** Boxplot p53 binding specificity control ChIP of the ChIP shown in Figure 3f performed in p53^-\-^ KP^fl^C cells (n=2).

**Supplementary Figure 5. Expression level of RNA-seq-identified genes in PDAC patients**. Expression level of a subgroup of genes identified in the RNA-seq from Figure 2 in p53 mutant and p53 wt cohorts of PDAC patients from TCGA dataset.

**Supplementary Figure 6 and 7. Full membrane images of the western blot data reported in main figures.**

**Supplementary Table1**:

| **q-PCR primers** | | |
| --- | --- | --- |
| **Gene** | **Forward primer** | **Reverse primer** |
| trp53 | TGAACCGCCGACCTATCCTTA | GGCACAAACACGAACCTCAAA |
| ube3c | TGTGTCTGAGGAACGACAGC | CCAGAAATGGACCATCGCCT |
| foxj1 | CTCTGACCGTGAACAGGGAG | CCTGGGTCAGCAGTAAGGTG |
| per2 | ATGATCCCAGACACGGAGGA | CTTCAACACCGCCTGGAGAT |
| arid3a | GAATGCCCGGATGCATAGGA | TCATCCCCAGAGATGGGAGG |
| hist2h2b3 | GCGGTCTACAATCACATCGC | CTACTTGGCGCTGGTGTACT |
| mst1r | CACGTGATGCTTCGATGCTG | GTGAGGCCACTACCTGCTTT |
| rcan2 | CACAGCCTGCCAAACAGTTC | TCCAGCGTGCAGCTCATATT |
| prex1 | GGCTGCCTGGAACATCTCTT | CATGGGGAAATGCCTGGTCT |
| il1rn | CCAGCTCATTGCTGGGTACT | AGACTTGGCACAAGACAGGC |
| inpp5j | ATCCTGGCTCCGAAGTCTCT | AATGCTGGCTTAGGTCCCAC |
| cd34 | GGTCACCTCTGGAGTTCTGC | GCCTTTCTCCTGTAGGGCTC |
| tp53 | CCCAAGCAATGGATGATTTGA | GGCATTCTGGGAGCTTCATCT |
| Mst1 #1 | AAGGATCCAAGTCACAGCGG | CCGCAGAGGTGGTATTGGTT |
| Mst1 #2 | TGCTATACCTTGGACCCGGA | CAGAATGGATGGTGGCTGGT |
| **Chip primers** | | |
| **Gene** | **Forward primer** | **Reverse primer** |
| trp63 | CCTCCTTAACGACACAGCCC | ACTTGAGAAGGAAAGGAGCCTG |
| nfya | TGGCCGATTCCACTCCATTT | AGACATTTCTGGACACTGGGG |
| ets-2 | GGAAAGCCCTGCACTTAGCA | CTAGCCACACAGTCCGTGC |
| srebf1 #1 | TAACCCTGCCAGCACAACTT | AGTAGAACTGGTCGGCAAGC |
| srebf1 #2 | TAGGAGGTCTCACAGCTGGTATT | CCTAGCCCCTCAGTAGCGT |
